# Genetic and Immune Changes Associated with Disease Progression under the Pressure of Oncolytic Therapy in A Neuroblastoma Outlier Patient

**DOI:** 10.3390/cancers12051104

**Published:** 2020-04-28

**Authors:** Lidia Franco-Luzón, Sandra García-Mulero, Rebeca Sanz-Pamplona, Gustavo Melen, David Ruano, Álvaro Lassaletta, Luís Madero, África González-Murillo, Manuel Ramírez

**Affiliations:** 1 Children Oncohematology Foundation, 28079 Madrid, Spain; lfluzon@gmail.com (L.F.-L.); luis.madero@salud.madrid.org (L.M.); 2 Department of Clinical Sciences, Faculty of Medicine and Health Sciences, University of Barcelona, 08036 Barcelona, Spain; s.garciam@idibell.cat; 3 Unit of Biomarkers and Susceptibility, Oncology Data Analytics Program (ODAP), Catalan Institute of Oncology (ICO), Oncobell Program, Bellvitge Biomedical Research Institute (IDIBELL) and CIBERESP, L’Hospitalet de Llobregat, 08908 Barcelona, Spain; rebecasanz@iconcologia.net; 4 Biomedical Research Foundation, Niño Jesús Children Hospital, 28009 Madrid, Spain; gustavo.melen@salud.madrid.org (G.M.); africa.gonzalez@salud.madrid.org (Á.G.-M.); 5 La Princesa Institute of Health Research, 28006 Madrid, Spain; druano64@hotmail.com (D.R.); lassaalvaro@yahoo.com (Á.L.); 6 Oncohematology Unit, Hospital Infantil Universitario Niño Jesús, 28009 Madrid, Spain

**Keywords:** neuroblastoma, oncolytic virotherapy, T lymphocytes (TILs), bioinformatic analysis, immune landscape

## Abstract

Little is known about the effect of oncolytic adenovirotherapy on pediatric tumors. Here we present the clinical case of a refractory neuroblastoma that responded positively to *Celyvir* (ICOVIR-5 oncolytic adenovirus delivered by autologous mesenchymal stem cells) for several months. We analyzed samples during tumor evolution in order to identify molecular and mutational features that could explain the interactions between treatment and tumor and how the balance between both of them evolved. We identified a higher adaptive immune infiltration during stabilized disease compared to progression, and also a higher mutational rate and T-cell receptor (TCR) diversity during disease progression. Our results indicate an initial active role of the immune system controlling tumor growth during *Celyvir* therapy. The tumor eventually escaped from the control exerted by virotherapy through acquisition of resistance by the tumor microenvironment that exhausted the initial T cell response.

## 1. Introduction

Our group is developing a unique strategy to deliver an oncolytic adenovirus (ICOVIR-5) [1,2,3] using autologous bone marrow-derived mesenchymal stem cells (MSC) in repeated intravenous administration in children and adults with advanced tumors. We named this new advanced therapy medicine (ATM) *Celyvir*, and we recently reported results of the first in human, first in children clinical trial [4,5]. *Celyvir* is a well-tolerated therapy, with very low or no toxicity, that can produce clinical responses in some patients, including children with advanced neuroblastoma.

ICOVIR-5 is an oncolytic adenovirus developed by Dr. Alemany and colleagues [1,2]. ICOVIR-5 (HAd5-DM-E2F-K-Δ24-RGD) is derived from human adenovirus serotype 5 (HAd5) and includes various genetic modifications that render its replication conditioned to the presence of a deregulated retinoblastoma pathway (pRb pathway) in tumor or malignant cells.

Clinical experiences with oncolytic adenoviruses are scarce [6,7,8], more so when considering systemic and repeated administrations like *Celyvir*. Little is known about important aspects of this new therapy: Pharmakokinetics (PK) and Pharmakodinamics (PD), capacity for tumor homing and barriers to reach them, kinetics of antiadenoviral immune responses of patients, among others. We and others have studied some of these crucial events in preclinical models [1,3,9,10], but we are in much need for information coming from patients.

Virotherapy is considered a form of immunotherapy [11,12,13,14] and so another important aspect of oncolytic adenoviruses in the clinical setting is to understand how viral replication in tumors might activate an antitumor immune response, how therapy coexists with the antiviral immune response, what impact therapy has on tumor immunology, including tumor infiltrating immune cells and the tumor microenvironment.

One patient with metastatic and refractory neuroblastoma was eligible for this study. The patient—aged 10—presented a neuroblastoma resistant to three lines of previous therapy (COJEC, E-SIOP, HR-NBL). Then the patient received *Celyvir* as sole therapy and showed an exceptional lasting response. We obtained biopsies of the primary tumor 4 and 20 months after initiating *Celyvir* therapy, when the disease was stabilized and eventually progressing, respectively. Clinical details of the patient were previously reported [4].

Outlier survivors of incurable cancers may offer unmatched opportunities for uncovering biological information of the disease that may help in designing better treatments for regular patients [15,16]. We present here results of a multi-omic analysis of primary tumor samples obtained at disease stabilization during oncolytic adenoviral therapy and at final tumor progression. Our study may help in understanding the process of tumor escape from the initial control exerted by adenovirus virotherapy.

## 2. Results

### 2.1. The Landscape of Infiltrating Immune Cells during Tumor Evolution under Oncolytic Virotherapy

We initially reported results of a cohort of patients with relapsed-refractory neuroblastoma that received weekly infusions of bone marrow-derived autologous mesenchymal cells carrying an oncolytic adenovirus as only therapy. Here we present an in-depth characterization of the patient that received the maximum doses of oncolytic virus (70 doses) [4].

**Figure 1:**
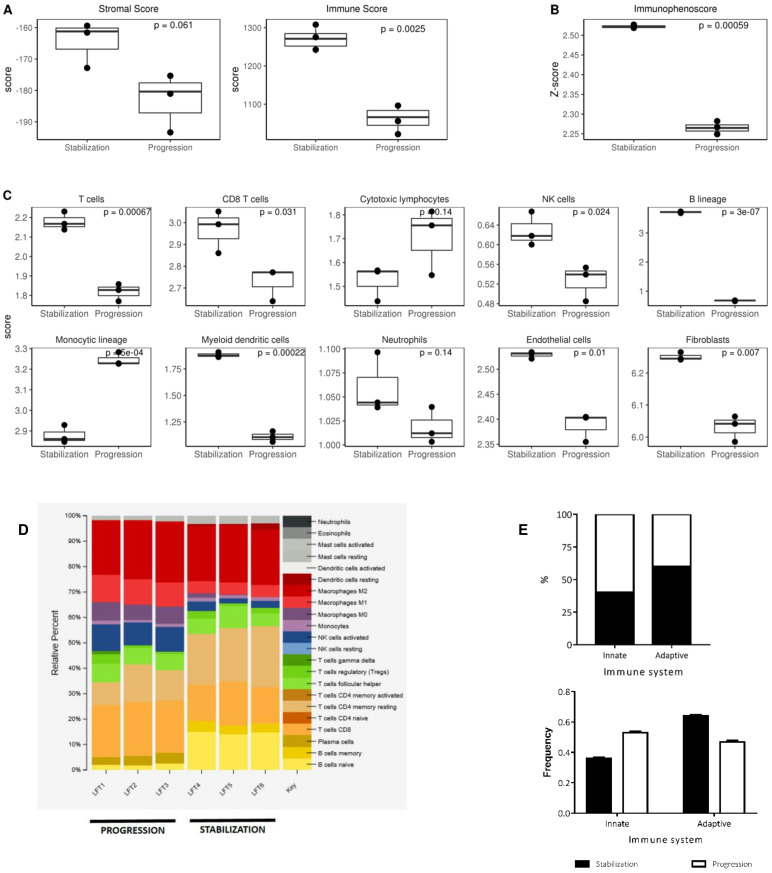
Immune cell estimation in tumor samples. (**A**) ESTIMATE (Estimation of STromal and Immune cells in MAlignant Tumor tissues using Expression data) graphs showed significant higher values of immune cells (*p* = 0.0025) were found in stabilized disease compared to progression, whereas no significant differences in stromal component were found between both samples (*p* = 0.061). (**B**) Immunophenoscore also showed higher number of immune cells during stabilization. (**C**) MCPcounter graphs showed the abundance of distinct immune subpopulations in both tumor samples. (**D**) CIBERSORT showed the proportions of distinct immune cell subpopulations. (**E**) Graphs of the main immune component of each sample.

RNA-Seq data obtained from tumor samples at disease stabilization during therapy and at final disease progression were analyzed using different algorithms, in order to ascertain biological characteristics of tumor evolution during oncolytic virotherapy pressure. Presence of infiltrating stromal/immune cells in tumor tissues was evaluated using ESTIMATE (Estimation of STromal and Immune cells in MAlignant Tumor tissues using Expression data) [17]. Major differences were found between immune score (*p* = 0.0025) and stroma score (*p* = 0.06, Figure 1A) at both stages of the disease. We found the stabilized disease was more infiltrated by immune cells compared to progression stage. Also, the Immunophenoscore, a measure of the overall immunogenicity of the tumor, was higher in stabilization than in progression (*p* = 0.0005, Figure 1B). Next, MCPcounter software (https://omictools.com/mcp-counter-tool) was used to obtain information about specific cell lineages infiltration. A predominance of B lymphocytes (score 3.5 vs. 0.5; *p* = 0.0000003), T lymphocytes (score 2.2 vs. 1.8; *p* = 0,0007), CD8 T cells (score 3 vs. 2.8; *p* = 0.0313), NK lymphocytes (score 0.6 vs. 0.55; *p* = 0.0241) and myeloid dendritic cells (score 1.8 vs. 1.1; *p* = 0,0002) was observed during stabilization. In contrast, monocytes were significantly more abundant during progression (score 3.2 vs. 2.9; *p* = 0,0005) compared to stable disease. Scores for endothelial cells and fibroblasts were lower at progression compared to stable disease (Figure 1C). The estimation of immune populations was also done using the QuanTIseq algorithm [18]. QuanTIseq analysis confirmed the presence of significantly more B cells (*p* = 0.011), dendritic cells (*p* = 0.024), NK cells (*p* = 0.026), and T lymphocytes (*p* < 0.05) during stabilization compared to progression. QuanTIseq also showed significantly higher abundance of M2 macrophages (*p* = 0.023) and a trend towards higher abundance of Tregs (*p* = 0.069) during stabilization, classically associated to a less inflamed and more protumoral tumor microenvironment (Supplementary Figure S1). We next estimated the relative abundance of 22 immune cell subtypes in each sample by CIBERSORT [19]. We identified B lymphocytes (naïve B cells and memory B cells) as the dominant population during disease stabilization. T CD4 memory predominated over CD8 within tumor infiltrating T lymphocytes (TILs) at that time, while M2 macrophages were the principal subpopulation among myeloid cells. During disease progression plasma cells appeared as the main component of B lymphocytes, while CD8 predominated over CD4 among TILs. Activated NK lymphocytes also appeared more represented at this time, while M2 macrophages predominated among the myeloid compartment, with increasing proportions of M0 and M1 macrophages (Figure 1D). In summary, the results of all analysis showed that a higher infiltration and activity of cells of the adaptive immunity dominated the immune landscape during oncolytic stabilization of the disease, evolving towards a more prominent presence of cells of the innate immunity when the tumor eventually progressed out of the control of the oncovirus therapy (Figure 1E).

We observed the significant higher presence of B lymphocytes in stabilized state compared to progression state (Figure 1C). However, distinct B lymphocytes subpopulations were overrepresented in both tumor samples when analyzed with CIBERSORT tool (Figure 1D). Naïve B cells were the most abundant population during stabilization, followed by memory B cells, whereas plasma cells were the only B cell subpopulation represented during progression. B cells played an active role during all disease stages, though we did not perform more deep analysis for these lymphocytes and we focused our analysis in T lymphocytes, more representative in previous literature. However, we find these results very interesting for future considerations regarding tumor infiltrating leukocytes studies.

**Figure 2:**
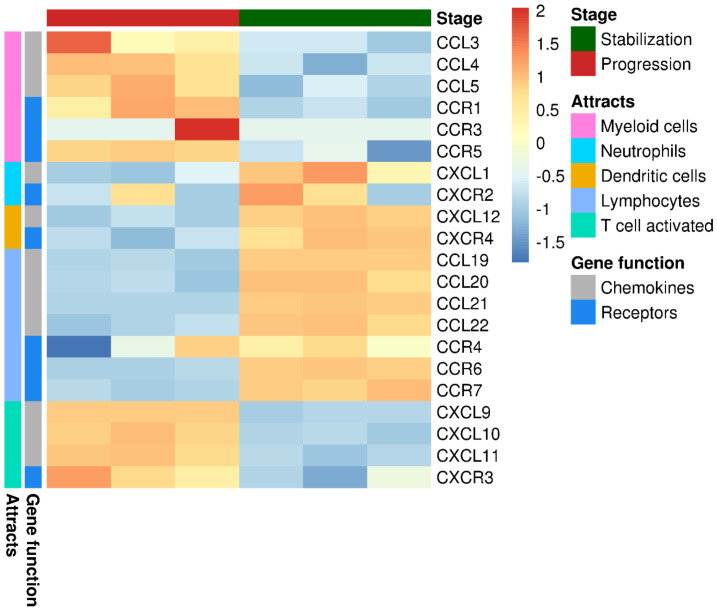
Heatmap showed correlations and abundance of distinct sets of chemokines at different stages of the disease. Chemokines related to lymphocytes, dendritic cells, and neutrophils were overrepresented during stabilization stage. However, chemokines related to both activated T cells and myeloid cells appeared to have a higher representation during progression.

We interrogated the data set to investigate changes in the profile of chemokine expression during tumor evolution that might correlated with the differences found in the immune cell infiltration already described. We found significantly higher expression of genes related to B lymphocytes chemotaxis (CCL [C-C motif Ligand]19, CCL21, CXCL [C-X-C motif) ligand]12, and CXCL13) during disease stabilization. Known lymphocytes (CCL19, CCL20, CCL21, and CCL22) and DCs (CXCL12) chemokines were also expressed at significantly higher levels at disease stabilization, while chemokines that recruit myeloid cells (CCL3, CCL4, CCL5), Treg (CCL4) and activated T lymphocytes (CXCL9, CXCL10, and CXCL11) were expressed at significantly higher levels during tumor progression (Figure 2).

### 2.2. Tumor Infiltrating T Lymphocytes during Tumor Evolution

**Figure 3:**
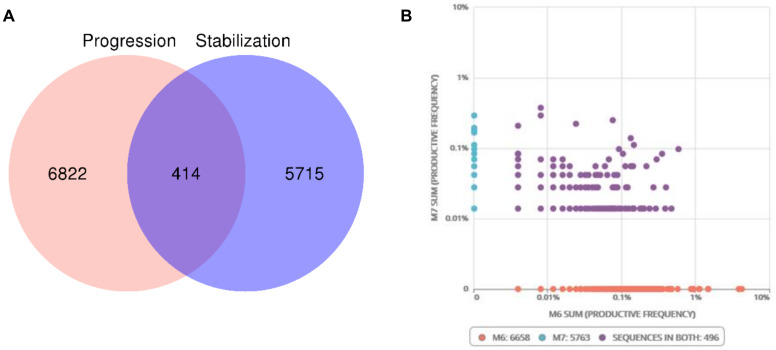
T-cell receptor (TCR) profile at both stages of the disease. (**A**) Total number of clonal rearrangements was higher during progression (M6) compared to stabilization (M7). Some of the rearrangements (414) were shared between both stages. (**B**) The abundance of rearrangements was higher during progression. Of these total number of rearrangements, 496 were present at both stages of the disease.

We next focused our analysis on tumor infiltrating T lymphocytes, a population containing antitumor T cells. Deep sequencing of T-cell receptor (TCR)beta chain gene analysis showed higher numbers of clonal rearrangements coming from the tumor sample obtained during the final progression of the disease (Figure 3A). In addition, the relative frequencies of the 10 most abundant rearrangements related to the pool of sequences were also higher during tumor progression (Supplementary Table S1). This suggests a more diverse TIL infiltration during disease evolution with fewer clones dominating the TIL landscape at the time of progression. Fifty percent of the 10 most abundant rearrangements found at the time of disease stabilization remained during progression. In total, 8% of the sequences present during stable disease also appeared in the tumor during progression, indicating the persistent infiltration by the same T cell clones (Figure 3B).

**Figure 4:**
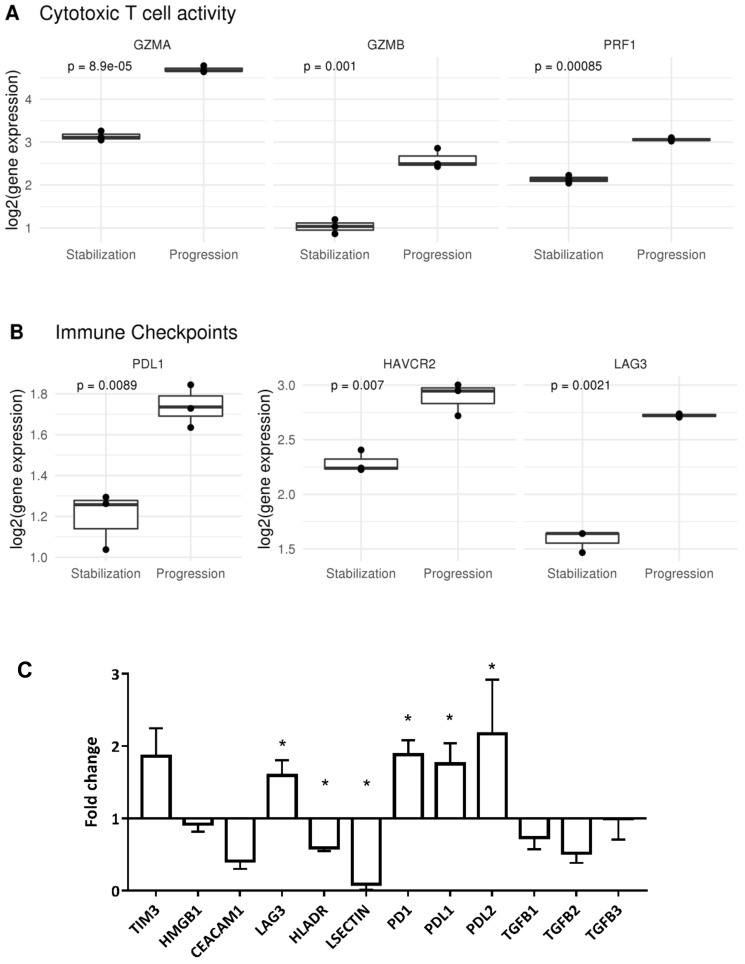
T lymphocytes activation / state phenotype was studied. (**A**) Granzimes and perforins showed an increased presence during progression stage, indicating a more activated T lymphocyte phenotype. (**B**) Exhaustion markers for T lymphocytes were also increased during progression. (**C**) Molecules associated to T cell exhaustion and immunosurveillance were analyzed by qPCR in both samples. Bars represent gene expression at the time of clinical progression. The result was normalized to the values of the sample corresponding to clinical stabilization (* *p* < 0.05). Fold change indicated how many times gene expression was higher/lower during progression compared to stabilization. During progression, exhaustion markers TIM3 (HAVCR2), LAG3, and PD1 were significantly overexpressed.

We analyzed the expression levels of genes related to T lymphocyte activation. T lymphocytes expressed higher levels of granzymes (GZMA, GZMB) and perforin (PRF1) genes (Figure 4A) at the time of progression, as well as markers associated to chronic activation (HAVCR2, LAG3, and TIGIT) (Figure 4B). We validated these findings by quantitative PCR for the T cell receptors related to T cell exhaustion (HAVCR2/TIM3, LAG3, and PD1), and the two most common ligands for each of them (HMGB1 and CEACAM; HLA-DR and LSECTIN; and PDL1 and PDL2, respectively), as well as the three known isoforms of TGFβ (TGFB1, TGFB2, and TGFB3). The results indicated that the microenvironment during tumor progression showed significantly higher expression levels for the ligands of the PD1 receptor (PDL1 and PDL2) compared to previous tumor stabilization status. LAG3 receptor was also significantly overexpressed in progression, but its two ligands (HLA-DR and LSECTIN) were significantly downregulated (Figure 4C). Therefore, tumor infiltrating T lymphocytes during progression showed higher expression of receptors related to T cell exhaustion in a tumor microenvironment that provided higher levels of the corresponding ligands. 

We also analyzed the genetic and molecular programs activated during disease using GSVA (*Gene Set Variation Analysis*). We found caspase/apoptosis pathways and cell cycle activation were higher during stabilization (Supplementary Figure S2).

### 2.3. Mutational and Neoepitope Landscape during Tumor Evolution

**Figure 5:**
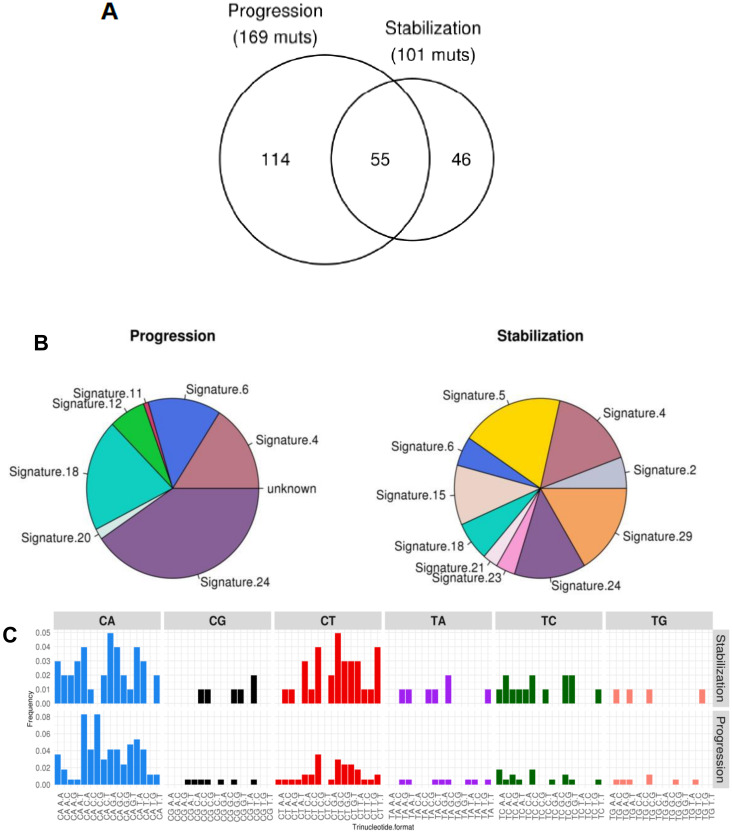
Mutational studies of both tumor samples. (**A**) Total number of mutations was higher during progression. (**B**) Diagrams showing the presence of different mutational signatures associated to different types of cancer in both tumor samples. (**C**) Diagrams showing the abundance of each base pair for both tumor samples. This distribution allowed to know the mutational signatures described in B.

We next interrogated our WES data sets to find out variations in the sequence of each tumor sample, looking for the mutational landscape. The total number of sequence variations found at progression was higher than those detected during disease stabilization, 169 versus 101. Overall, a total of 55 single nucleotide variations were common to both samples (Figure 5A). CONDEL software [20] was used to identify genetic mutations with a putative functional impact. Table 1 lists mutations identified in the sample corresponding to tumor progression that were absent during stabilization. 

COSMIC (Catalog of Somatic Mutations in Cancer) mutational signatures was inferred from WES data. Results indicated the unique and common genetic signatures for each sample, as well as their relative frequencies (Figure 5B, Table 2). Data was extrapolated from the abundance of each base (A, T, G, or C) in the genome of each sample (Figure 5C). There was greater signature variability during stabilization compared to progression state. However, relative signature abundances of the progression are greater (especially patent in signature 24). Of special interest were two of the signatures identified: Signature 18, which is classically associated with neuroblastoma and appears during the progression of the disease, and signature 2, which appears during stabilization state and is associated to a wide variety of cancers, and was related to the activation of AID/APOBEC deaminase.

Regarding common mutations in neuroblastoma, both tumors at time of stabilization and progression harbored *ATRX* mutation. We used NetMHC [21,22] to predict neoepitopes based on the presence of single nucleotide variants (SNVs) detected in each tumor sample and patient’s human leukocyte antigen (HLA). This analysis revealed a higher neoepitope candidate load in the tumor at progression compared to that during stable disease, 18 versus 5. Interestingly, all 5 candidates found during disease stabilization were also detected at progression. (Table 3 and Table 4).

**Figure 6:**
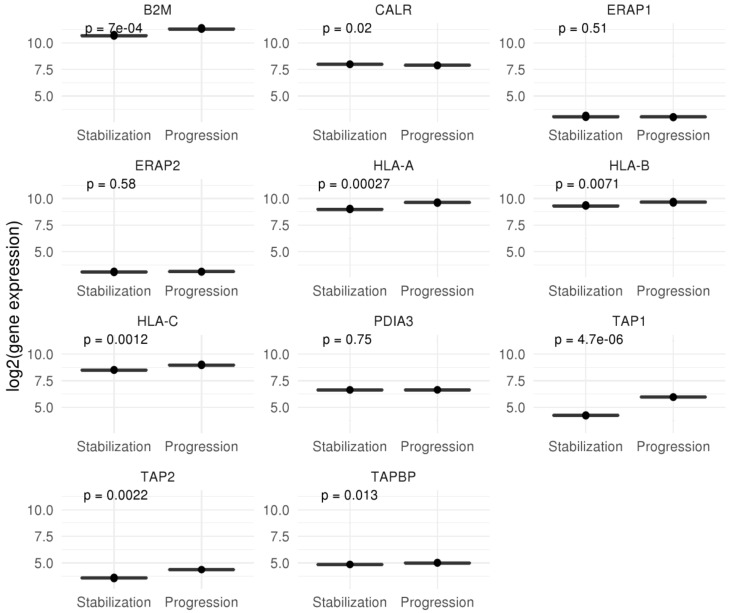
Gene expression of molecules related to antigen presentation and processing, including HLA molecules.

Once identified neoepitope candidates, we studied the integrity of genes related to antigen processing and presentation. RNA-Seq analysis showed no deficit in the expression of antigen processing and presentation. In fact, expression of most of these genes was higher at progression compared to stabilized disease (Figure 6). These results suggest that the neoepitopes could be expressed in HLA molecules at any time during tumor evolution. 

## 3. Discussion

We present here genomic studies of tumor samples at different moments during systemic virotherapy in a case of refractory neuroblastoma. The patient may be classified as outlier based on her clinical outcome; she survived for 22 months with metastatic neuroblastoma refractory to 3 lines of therapy, receiving oncolytic virotherapy as sole treatment. Although limited for coming from a single case and the lack of functional validation, the information may help in understanding the process of tumor escape from the control exerted by virotherapy, for which scant information currently exists [23,24]. The snapshots taken at two different time points of the evolution of this tumor showed striking differences that corresponded to different biological behavior and clinical responses. Both tumor cells and infiltrating immune ones transited from disease stabilization to progression by accumulating changes in many aspects of their biology. 

We did not find evidence that virotherapy exerted significant tumor lytic effect during disease stabilization. We do not have data on the antiadenoviral antibody titers for the patient, therefore we ignore the effects of a likely immune response against the oncolytic adenovirus. However, we have previously shown in mouse [25] and in dogs [26] treated with species-specific oncolytic adenoviruses that the presence of a humoral immune response does not prevent the antitumor effect of the virus. Detection of adenovirus in tumor samples by highly sensitive real-time PCR showed minimum amount of virus at both time (data not shown). All measurable tumor was detectable at any time from starting virotherapy. As already commented in a first report [4], neuroblastoma cells were mainly in a quiescent state during disease stabilization, with no signs of tumor lysis. We show here that the presence of more proliferative tumor cells at progression was associated with higher numbers of genetic alterations in the tumor genome at that time. One third of these mutations were already present during stabilization, when they contributed to more than half of the total mutational burden. Acquisitions of new mutations during disease progression are expected in the context of an active neoplasia that maintained the intrinsic genetic instability of human tumor cells [27,28,29,30,31]. Among all mutated genes identified only at progression, CREG1 (*Cellular repressor of E1A stimulated genes 1*) is particularly attractive as a candidate for validation because it represses the activity of the adenovirus E1A protein, and also controls the activation and repression of pathways that induce proliferation and inhibit differentiation [32,33,34]. 

In parallel, the total number of possible neoantigens was higher comparing progressive tumor to stabilized one. Expression of these putative neoepitopes was confirmed at transcription level. Moreover, genes related to antigen processing and presentation appeared unaffected in any sample, in fact we detected higher level of expression in the sample corresponding to tumor progression. Therefore, it seems likely that tumor associated antigens could be efficiently presented to cells of the adaptive immune branch at any time during disease evolution. 

Analysis of gene data sets with different algorithms estimated a decreasing proportion of tumor infiltrating immune cells from disease stabilization to progressing tumor. More important than changes in the relative number of immune cells, the contribution of individual subpopulations also varied with time. Cells of the adaptive immune system dominated the immune infiltration landscape during oncolytic stabilization of the disease, evolving towards a more prominent innate immunity when the tumor eventually progressed out of the control of the oncoadenovirus therapy. The presence of the major immune cell populations also corresponded to the chemokine profiles responsible for their recruitment, as was the case with those related to B lymphocytes and dendritic cells at the time of disease stabilization [35,36]. 

CIBERSORT analysis showed that M2 were the most frequent subtype among macrophages at both time points, however, during progression the subpopulations of non-polarized and M1-polarized macrophages increased compared to stable disease. This change suggests an increased recruitment of uncommitted macrophages to the tumor mass during tumor progression, with eventual differentiation towards M1 and, predominantly, M2 subtypes. All these myeloid cell populations have been related to a more pro-tumoral environment [37,38,39,40].

Within the infiltrating T lymphocytes, we found more diversity of TCRs at time of tumor progression than during stabilization. Fewer clonal TCR rearrangements dominated among total TCRs at the end compared to the previous stage. The most frequent TCR rearrangements found during stable disease were also detected at the end stage. Since we could not do functional studies to assess the presence of tumor reactive T lymphocytes, we do not know the reactivity of these TCR clone sequences. It has been reported that many non-tumor-specific T lymphocytes infiltrate human tumors [41,42], so we cannot conclude that the wider variety of TCRs found in the progressing tumor correspond to higher antitumor infiltrating T cells. It is worth noticing though that higher mutational and neoepitopes load during tumor evolution were associated to a greater variety of TCRs, even in a shrinking population of TILs. Furthermore, TCR diversity in tumor tissue could be associated to poor prognosis in some cases [43,44]. The chemokine environment showed enrichment in molecules related to the recruitment of activated T lymphocytes during progression. We found that granzymes and perforins were more abundant during disease progression compared to stabilization stage. These cytolytic effector molecules are associated to T cell and NK cell cytolytic activity [45,46,47]. T lymphocytes that infiltrated the tumor in the final phase of the disease showed higher expression levels of chronic activation markers (PD1, LAG3) [48,49], while ligands for PD1 were significantly overexpressed in the tumor microenvironment at progression. This suggests that despite immune infiltration by T lymphocytes, intratumor conditions at progression favored T cell exhaustion [50,51,52].

Marked decrease in the B cell compartment from stabilized to progressing tumor was also seen, suggesting that B lymphocytes could have also participated in tumor control through production of antibodies and complement cascade, or recruiting DC through CXCL12 secretion [53,54].

In summary, oncolytic adenoviral therapy could not eliminate the tumor in our patient but certainly exerted some control over it and prevented its progression for an exceptional long period through immune-related mechanisms. Repeated administration of oncolytic virotherapy initially induced local immune infiltrates dominated by adaptive cells, and the patient obtained the stabilization of a, so far, refractory disease. The pressure exerted by therapy along time eventually selected the acquisitions of resistance mechanisms such as recruitment of myeloid cells into the tumor microenvironment and upregulation of T cell exhaustion inducing molecules. In order to improve results and prevent tumor escape, we foresee the combination of *Celyvir* with additional synergistic strategies: Radiotherapy (enhances MSCs into irradiated areas [55]), chemotherapies that do not cause lymphodepletion [56], or the use of checkpoint inhibitors, as it has already been reported in patients with melanoma treated with oncolytic virotherapy [57]. 

## 4. Materials and Methods 

### 4.1. Patient’s Samples

Fresh tumor samples were obtained from surgery in two different moments of disease stage and tumor development. The first biopsy was obtained when the patient still responded to oncolytic virotherapy treatment (stabilization sample), while the second one was obtained during disease progression (progression sample). Both of them were stored at −80 ºC until their processing for this study. From each sample we isolated RNA and DNA separately.

### 4.2. gDNA Isolation and Quantification

For gDNA isolation, we used Qiamp DNA Mini Kit (Qiagen, Hilden, Germany). Three samples were processed to obtain gDNA. Stabilization sample, progression sample, and control sample. Stabilization and progression samples corresponded to response to treatment and progression disease, respectively. Control sample consisted on healthy mesenchymal stem cells (MSC) from the patient. Tissues or cells were digested with Proteinase K and then processed with reagents from the kit. gDNA was measured using Thermo Scientific NanoDrop 1000 spectrophotometer (Thermo Fisher Scientific, Wilmington, DE, USA). Total gDNA form each sample was diluted in DNAse free water and sent to Sistemas Genómicos S.L. (Valencia, Spain) to perform Next Generation Sequencing (NGS) studies.

### 4.3. Whole Exome Sequencing (WES): Variant Calling and Mutational Signatures

WES data was analyzed according to GATK best-practices guidelines [58,59]. FastQC software [60] (consult online in: https://www.bioinformatics.babraham.ac.uk/projects/fastqc/) was used to assess reads quality. Trimmomatic software [61] was used to make a trimming of the first and second base, due to bad quality. Then, reads were mapped over reference human genome (hg19/GRCh38) using BWA MEM tool [62]. A base recalibration realignment and a removal of duplicates were done using Picard. Variant calling was done with Mutect1 software [59] using a normal paired sample as a reference to filter polymorphisms. Variants with frequency >10 and DP > 20 were selected and annotated using Annovar software [63]. The contribution of COSMIC [64] mutational signatures was calculated with the R package deconstructSigs [65]. Detailed and complete list of each different genetic signatures can be consulted online [64] (https://cancer.sanger.ac.uk/cosmic/signatures_v2). In this case, the study focused on analyzing each signature of those present in the patient’s tumor tissues. Total mutation burden was estimated by considering single nucleotide variants (SNV) from exonic regions. The pathogenicity of the identified missense variants was analyzed by using the metapredictor CONDEL [20].

### 4.4. T-Cell Receptor (TCR) Sequencing

TCR Sequencing was performed with immunoSEQ Assay by Adaptive Technologies (adaptivebiotech.com). Their sequencing protocols are based in a multiplex PCR amplification directly from genomic DNA, which allow to sequence and identify CDR3 chains highly represented in the population of T cells found in the sample. The full protocol was developed by Carlson and colleagues [61]. Graphs were generated using immunoSEQ Analyzer.

### 4.5. Neoantigen Prediction

SNV annotated as missense were used for epitope prediction purposes. To do this, human FASTA sequences extracted from UniProt were used to translate information about DNA missense mutations into an amino acid change level (only isoforms 1 were taken into account). For each missense mutation, a sequence of 19-amino acids centered on the mutation were analyzed for potential neoantigens. NetMHCCons was used to infer putative immunogenic peptides (19 aa; 9 mer) combining information about HLAs patient’ genotype and peptides harboring missense mutations. An immunogenic epitope was defined as a mutated peptide with high affinity for one HLA allele IC50 < 50nM.

### 4.6. RNA Isolation and Quantification

Both samples were minced using a Tissue Homogenizer (VDI 12, VWR International Ltd, Leicestershire, England, UK) and tissue RNA isolation was performed using RNeasy Plus Mini Kit (Qiagen) which includes genomic DNA elimination columns. RNA was quantified with Thermo Scientific NanoDrop 1000 spectrophotometer (absorbance at 260 nm and the ratio of 260/280 and 260/230). Aliquots of 5 µg of total RNA were prepared to send in a final volume of 50 µL of RNAse free water. Both samples were sent to Biotechvana (Parque Científico de Madrid, Spain) for its analysis.

### 4.7. RNA-Seq Analysis: Expression Matrix, Differentially Expressed Genes, and Functional Analysis

Samples were processed by Biotechvana company and studies were performed in Unit of Biomarkers and Susceptibility (ICO-Idibell).

From each sample we obtained 3 technical replicates to work with (6 libraries from 2 samples in total). FastQC software was used to assess reads quality. To remove Illumina adaptors, a trimming of these sequences was done using Trimmomatic software. Then, reads were mapped over reference human genome (hg19/GRCh38) using STAR tool [62]. An annotation file in GTF format (downloaded from the UCSC Table Browser, using RefSeq [63] genes table including 23,687 genes and 41,970 transcript isoforms were used for the indexing step. Finally, RSEM tool [64] was used over aligned reads (BAM files) to extract a matrix of gene expression in terms of FPKM (Fragments per Kilobase per Million mapped fragments). We filter not expressed genes and finally we performed a TMM (trimmed mean of M-values) normalization to reduce variability across samples. 

To identify differentially expressed genes (DEG) between the two conditions, a linear model was fitted using the R package Limma [65]. A list of DEG with *p*-value < 0.01 and logFC > 1 was extracted. 

Then, the Gene Set Enrichment analysis (GSEA) algorithm [66] was used to identify enrichment in specific cellular functions and pathways.

### 4.8. Immune Profile Analysis

To analyze which immune populations were most abundant in each analyzed sample we used a variety of algorithms optimized for deconvolution of immune cell-types from RNA-Seq data. Tumor purity, stromal, and immune status were estimated using the R packages ESTIMATE [17] and Immunophenoscore [67]. RNA-Seq analysis data was used to obtain the three scores of ESTIMATE that give us idea of tumor purity and immune infiltration: stromal score (quantifies the presence of stroma in tumor tissue); immune score (that represents the infiltration of immune cells in tumor tissue) and estimate score (infers tumor purity). Immunophenoscore was then used to calculate the immune state of the samples.

Next, proportion of immune cell infiltration were calculated with QuanTIseq [18], CIBERSORT [19] and MCPcounter [68]. The online version of CIBERSORT (https://cibersort.stanford.edu/index.php) is able to analyze which immune populations are related to overexpressed genes obtained through RNA-Seq analysis. We crossed our RNA-Seq data from each sample with pre-settled LM22 leucocyte gene signature matrix.

To obtain a more detailed picture of immune cell-types infiltration, R package MCPcounter was used. MCP-counter (Microenvironment Cell Populations-counter) is a deconvolution method for quantification of immune cell’s relative abundances in heterogeneous tissues using marker genes. Nine different cell types were interrogated (T cells, Cytotoxic T cells, NK cells, B lineage, monocytic lineage, myeloid dendritic cells, neutrophils, endothelial cells, and fibroblasts). Results obtained were validated by the analysis with QuanTIseq, which performs an absolute quantification of cell types in the samples.

To analyze which immune populations were most abundant in each analyzed sample we used CIBERSORT. This online tool (https://cibersort.stanford.edu/index.php) [19] is able to analyze which immune populations are related to overexpressed genes obtained through RNA-Seq analysis. We crossed our RNA-Seq data from each sample with pre-settled LM22 leucocyte gene signature matrix. Obtained data is discussed on Results section.

### 4.9. GSVA: Gene Set Variation Analysis for Microarray and RNA-Seq Data

Chemokine profiles from each different sample were inferred through Gene Set Variation Analysis (GSVA) algorithm [69].

### 4.10. Statistical Analysis

For all the obtained scores, assumptions of normality and homoscedasticity were interrogated, and all comparisons between variables were analyzed using non-parametric tests (Wilcoxon test). For all tests applied, differences were considered significant when *p*-value < 0.05.

## 5. Conclusions

Extensive analysis of omic information obtained from samples gathered during the evolution of a neuroblastoma treated with oncolytic adenovirus helped in understanding the mechanisms of tumor escape from the control initially contributed by virotherapy. Tumor progression eventually selected the acquisitions of resistance mechanisms including recruitment of myeloid cells into the tumor microenvironment and upregulation of T cell exhaustion inducing molecules. 

## Figures and Tables

**Table 1:** List of mutations predicted by CONDEL for progression stage.

Gene	Description
*IFT140*	Intraflagellar transport 140
*DNASE1*	Deoxyribonuclease 1
*DNAH9*	Dynein axonemal heavy chain 9
*GALNT15*	Polypeptide N-acetylgalactosaminyltransferase 15
*ZNF98*	Zinc finger protein 98
*GABBR1*	Gamma-aminobutyric acid type B receptor subunit 1
*KIAA0391*	KIAA0391
*MIPOL1*	Mirror-image polydactyly 1
*RYR1*	Ryanodine receptor 1
*UCHL1*	Ubiquitin C-terminal hydrolase L1
*EML2*	Echinoderm microtubule associated protein like 2
*CELSR3*	Cadherin EGF LAG seven-pass G-type receptor 3
*ABHD2*	Abhydrolase domain containing 2
*RIPK2*	Receptor interacting serine/threonine kinase 2
*IQGAP1*	IQ motif containing GTPase activating protein 1
*VWA3B*	Von Willebrand factor A domain containing 3B
*AGL*	Amylo-alpha-1, 6-glucosidase, 4-alpha-glucanotransferase
*PABPC1*	Poly(A) binding protein cytoplasmic 1
*SLK*	STE20 like kinase
*ALDH2*	Aldehyde dehydrogenase 2 family (mitochondrial)
*GLE1*	GLE1, RNA export mediator
*LRP1B*	LDL receptor related protein 1B
*TRPV6*	Transient receptor potential cation channel subfamily V member 6
*ASIC5*	Acid sensing ion channel subunit family member 5
*SI*	Sucrase-isomaltase
*CREG1*	Cellular repressor of E1A stimulated genes 1
*PSMD1*	Proteasome 26S subunit, non-ATPase 1

**Table 2:** Mutational signatures detected in both tumor samples after quantification of DNA bases.

Stabilization		Progression	
Signature	Percentage	Signature	Percentage
Signature 5	19.1928%	Signature 24	40.5295%
Signature 29	16.4542%	Signature 18	20.1733%
Signature 4	15.7368%	Signature 4	15.8887%
Signature 24	13.4168%	Signature 6	13.7232%
Signature 15	10.7573%	Signature 12	6.8523%
Signature 18	7.0359%	Signature 20	1.9288%
Signature 2	5.6384%	Signature 11	0.9041%
Signature 6	5.289%		
Signature 23	3.6131%		
Signature 21	2.8656%		

**Table 3:** Detailed predicted neoepitopes in both stages of the disease. Predicted neoepitopes in stabilized disease.

Identity (Protein the Peptide Comes from)	Number of Times Identity Appears in Analysis	Description
ASIC5	2	Acid Sensing Ion Channel Subunit Family Member 5
YLPM1	1	YLP Motif Containing
SLC38A1	1	Solute Carrier Family 38 Member 1
HMGB3	1	High Mobility Group Box 3

**Table 4:** Predicted neoepitopes in progression disease.

Identity (Protein the Peptide Comes From)	Number of Times Identity Appears in Analysis	Description
OR2M2	5	Olfactory Receptor Family 2 Subfamily M Member 2
UCHL1	1	Ubiquitin C-Terminal Hydrolase L1
ASIC5	3	Acid Sensing Ion Channel Subunit Family Member 5
YLPM1	1	YLP Motif Containing
ZNF98	1	Zinc Finger Protein 98
AGL	1	Amylo-Alpha-1, 6-Glucosidase, 4-Alpha-Glucanotransferase
GHRL	2	Ghrelin and Obestatin Prepropeptide
GALNT15	2	Polypeptide N-Acetylgalactosaminyltransferase 15
CELSR3	1	Cadherin EGF LAG Seven-Pass G-Type Receptor 3
UCHL1	2	Ubiquitin C-Terminal Hydrolase L1
SLC38A1	1	Solute Carrier Family 38 Member 1
HMGB3	1	High Mobility Group Box 3

## Supplementary Materials

The following are available online at https://www.mdpi.com/2072-6694/12/5/1104/s1, Figure S1: Immune phenotypes predicted by QuanTIseq for both disease stages; Figure S2: Genomic and molecular pathways and processes related to apoptosis and cell cycle for both disease stages, Table S1: Ten most common rearrangements for each tumor sample.
