# Supplementary material for: Genetic and Immune Changes Associated with Disease Progression under the Pressure of Oncolytic Therapy in A Neuroblastoma Outlier Patient
Source: Cancers (Basel). 2020 Apr 28;12(5):1104. doi: 10.3390/cancers12051104 (PMC7281487; doi:10.3390/cancers12051104)
Supplement: Supplementary file 1 [file cancers-12-01104-s001.pdf]

Supplementary Materials: Genetic and Immune Changes Associated with Disease Progression under the Pressure of Oncolytic Therapy in A Neuroblastoma Outlier Patient

Lidia Franco-Luzón [1], Sandra García-Mulero [2,3], Rebeca Sanz-Pamplona [3], Gustavo Melen [4,5], David Ruano [5], Álvaro Lassaletta [5], Luís Madero [1,5,6], África González-Murillo [4,5] and Manuel Ramírez [4,5,*]

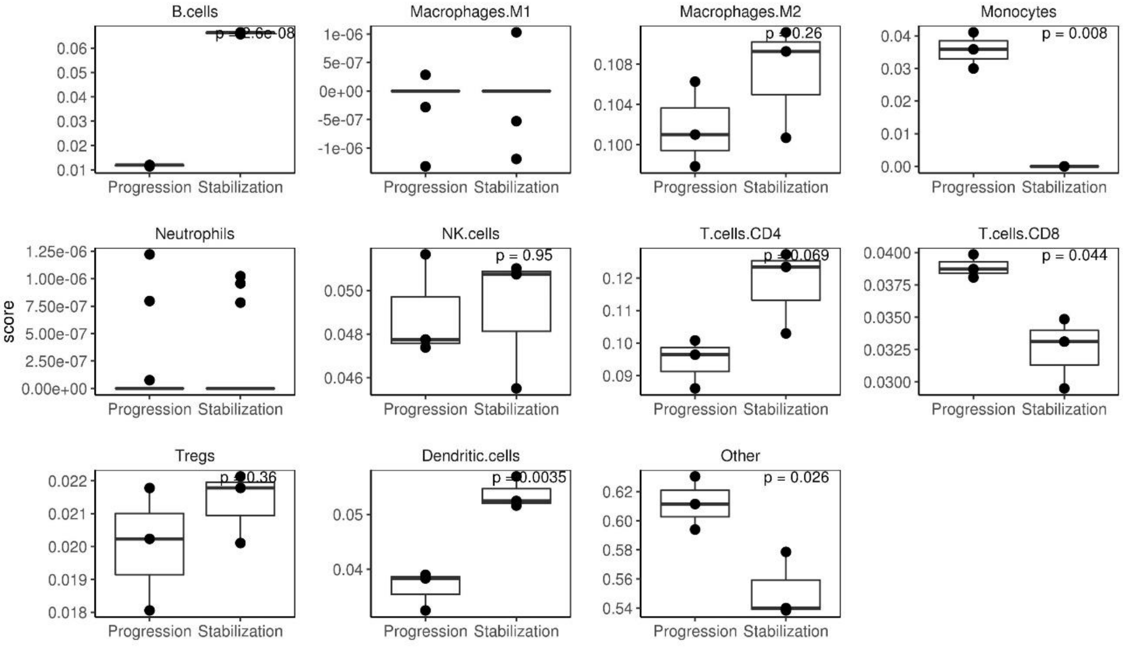

Figure S1. Immune phenotypes predicted by QuanTiSeq for both disease stages.

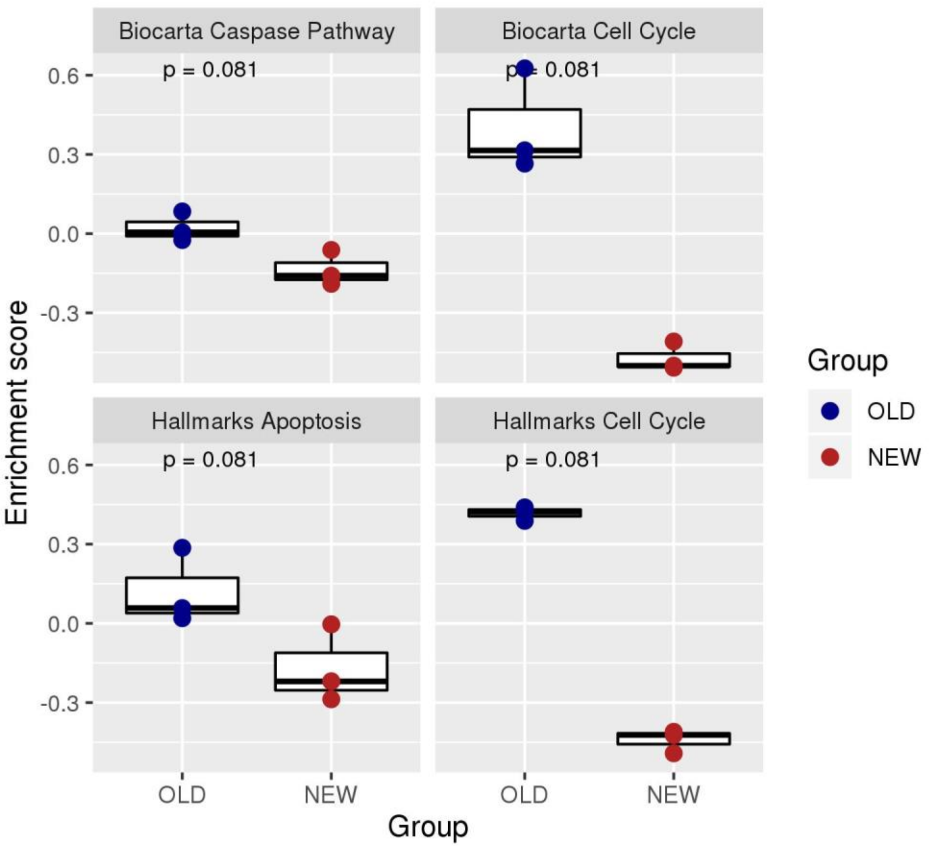

Figure S2. Genomic and molecular pathways and processes related to apoptosis and cell cycle for both disease stages.

Table S1. Ten most common rearrangements for each tumor sample.

Sample	Position	Present in:	Rearrangement	Relative frequencies
Disease progression stage (NEW sample)	1	1	CACGCCCTGCAGCCAGAAGACTCAGCCCTGTATCTCTGCGCCAGCAGCCAAGAGGGGCTGGGCTCCTACGAGCAGTACTTCGGGCCG	4.318456%
	2	1	AACGCCTTGGAGCTGGACGACTCGGCCCTGTATCTCTGTGCCAGCAGCTTGGTAATAGCCCGGTACAATGAGCAGTTCTTCGGGCCA	3.876156%
	3	1	CACGCCCTGCAGCCAGAAGACTCAGCCCTGTATCTCTGCGCCAGCAGCCAAGAGAGACTAGGAACCTACGAGCAGTACTTCGGGCCG	1.519903%
	4	1	CTGCTGGGGTTGGAGTCGGCTGCTCCCTCCCAAACATCTGTGTACTTCTGTGCCAGCAGTAAGAACACTGAAGCTTTCTTTGGACAA	1.451548%
	5	1	CGCACAGAGCAGCGGGACTCGGCCATGTATCGCTGTGCCAGCAGCTTAGGAAAAAGGCAGGGTAACACTGAAGCTTTCTTTGGACAA	1.145959%
	6	1	TCTGCCAGGCCCTCACATACCTCTCAGTACCTCTGTGCCAGCAGTAACCTCGGACTAGCGGGAACCGGGGAGCTGTTTTTTGGAGAA	1.125854%
	7	1	CTGGAGTCGGCTGCTCCCTCCCAGACATCTGTGTACTTCTGTGCCAGCAGGCCGACAGGGGGCGTCTACGAGCAGTACTTCGGGCCG	0.940893%
	8	1	CTCACTCTGGAGTCCGCTACCAGCTCCCAGACATCTGTGTACTTCTGTGCCACGAGATGGGGAGGCACTGAAGCTTTCTTTGGACAA	0.940893%
	9	1	CTGAGCTCTCTGGAGCTGGGGGACTCAGCTTTGTATTTCTGTGCCAGCAGCCCCCCCGCAGCAAACTATGGCTACACCTTCGGTTCG	0.8846%
	10	1	CTGCAGCCAGAAGACTCGGCCCTGTATCTCTGTGCCAGCAGCCAAGATTTGGCTAGCGGGAGCTTCGGGGAGCTGTTTTTTGGAGAA	0.583032%
Disease stabilization stage (OLD sample)	1	1	CTGACCCTGGAGTCTGCCAGGCCCTCACATACCTCTCAGTACCTCTGTGCCAGCAGTGACAGGGGGCAGGGCTACACCTTCGGTTCG	0.375313%
	2	1	TCTAAGAAGCTCCTTCTCAGTGACTCTGGCTTCTATCTCTGTGCCTGGAGTGCCGGGACAGGGAGGAAGACCCAGTACTTCGGGCCA	0.29191%
	3	1	CCGCTCAGGCTGGAGTTGGCTGCTCCCTCCCAGACATCTGTGTACTTCTGTGCCAGCACCGACAGCTATGGCTACACCTTCGGTTCG	0.29191%
	4	1	CTACACACCCTGCAGCCAGAAGACTCGGCCCTGTATCTCTGTGCCAGCAGCGGCCGGGACAGGGACTATGGCTACACCTTCGGTTCG	0.250209%
	5	1	ACTCTGAAGATCCAGCCCTCAGAACCCAGGGACTCAGCTGTGTACTTCTGTGCCAGCAGTTTCGGGGGGCAGGAGTACTTCGGGCCG	0.222408%
	6	1	GAGTCGGCTGCTCCCTCCCAGACATCTGTGTACTTCTGTGCCAGCAGTTCTGAGAGCTCCTATAATTCACCCCTCCACTTTGGGAAC	0.208507%
	7	1	CTGGAGTCGGCTGCTCCCTCCCAGACATCTGTGTACTTCTGTGCCAGCACGTACCGTGAATCAGGAGAGACCCAGTACTTCGGGCCA	0.194607%
	8	1	CCGCTCAGGCTGGAGTTGGCTGCTCCCTCCCAGACATCTGTGTACTTCTGTGCCAGCACCCCCGGCTATGGCTACACCTTCGGTTCG	0.194607%
	9	1	GTGAACGCCTTGTTGCTGGGGGACTCGGCCCTCTATCTCTGTGCCAGCAGCTCGGGGACTAGCGAGGGCGAGCAGTACTTCGGGCCG	0.180706%
	10	1	ACTCTGACAGTGACCAGTGCCCATCCTGAAGACAGCAGCTTCTACATCTGCAGTGGCCCTAGCGGGGGGACCCAGTACTTCGGGCCA	0.180706%
